# Ribosome heterogeneity and specialization in development

**DOI:** 10.1002/wrna.1644

**Published:** 2021-02-09

**Authors:** Karl Norris, Tayah Hopes, Julie Louise Aspden

**Affiliations:** ^1^ Faculty of Biological Sciences, School of Molecular and Cellular Biology University of Leeds Leeds UK; ^2^ Leeds Omics University of Leeds Leeds UK

**Keywords:** development, *Drosophila melanogaster*, mRNA translation, ribosomal protein, Ribosome

## Abstract

Regulation of protein synthesis is a vital step in controlling gene expression, especially during development. Over the last 10 years, it has become clear that rather than being homogeneous machines responsible for mRNA translation, ribosomes are highly heterogeneous and can play an active part in translational regulation. These “specialized ribosomes” comprise of specific protein and/or rRNA components, which are required for the translation of particular mRNAs. However, while there is extensive evidence for ribosome heterogeneity, support for specialized functions is limited. Recent work in a variety of developmental model organisms has shed some light on the biological relevance of ribosome heterogeneity. Tissue‐specific expression of ribosomal components along with phenotypic analysis of ribosomal gene mutations indicate that ribosome heterogeneity and potentially specialization are common in key development processes like embryogenesis, spermatogenesis, oogenesis, body patterning, and neurogenesis. Several examples of ribosome specialization have now been proposed but strong links between ribosome heterogeneity, translation of specific mRNAs by defined mechanisms, and role of these translation events remain elusive. Furthermore, several studies have indicated that heterogeneous ribosome populations are a product of tissue‐specific expression rather than specialized function and that ribosomal protein phenotypes are the result of extra‐ribosomal function or overall reduced ribosome levels. Many important questions still need to be addressed in order to determine the functional importance of ribosome heterogeneity to development and disease, which is likely to vary across systems. It will be essential to dissect these issues to fully understand diseases caused by disruptions to ribosomal composition, such as ribosomopathies.

This article is categorized under:

Translation > Translation Regulation
Translation > Ribosome Structure/Function
RNA in Disease and Development > RNA in Development

## Graphical abstract

Ribosome heterogeneity is found across a variety of species and its potential importance to development is being assessed to determine if these ribosomes are specialized.

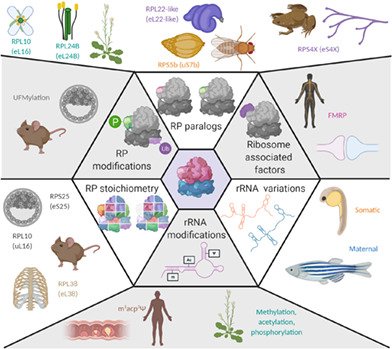

## 1 INTRODUCTION

The regulation of mRNA translation is essential to developmental processes and their control, from embryogenesis to neurogenesis, across a myriad of organisms. Although the main focus within developmental biology has been transcriptional control (Theunissen & Jaenisch, 2017), translational regulation is widespread and complex (Teixeira & Lehmann, 2019). In fact, there is poor correlation between mRNA and protein levels during key developmental time points, for example, *Xenopus* embryogenesis (Peshkin et al., 2015). mRNA translation can be regulated through a variety of well‐characterized mechanisms including: the binding of proteins to untranslated regions (UTRs), upstream open reading frames (uORFs), RNA structures within the mRNA and miRNA/lncRNA‐mRNA base pairing (Gebauer & Hentze, 2004; Tahmasebi et al., 2018). Until recently, the ribosome itself was not thought to be part of this regulatory system.

Once considered a homogenous macromolecular machine, recent evidence has revealed that ribosomes are heterogenous in their composition. This raises the exciting possibility that compositional differences in ribosome populations could provide a mechanism by which mRNA translation is regulated: different groups of ribosomes could be tailored to translate specific groups of mRNAs. These are termed specialized ribosomes.

The examination of phenotypes resulting from gene disruption is central to understanding gene function in organismal development. Specific phenotypes and human disease symptoms, resulting from ribosomal protein (RP) mutations, have been central to the evolution of the specialized ribosome theory. Dissecting the biological function of “specialized ribosomes,” particularly through development, will be essential in determining where different ribosome populations exhibit distinct functions and whether these are important to cellular and organismal health.

Here we review and explore the evidence for ribosome heterogeneity and specialization during development. In the last couple of years, work has been published in a number of key developmental model organisms [*Drosophila melanogaster* (Mageeney & Ware, 2019), *Caenorhabditis elegans* (Cenik et al., 2019), *Mus musculus* (Hebras et al., 2020), *Xenopus laevis* (Shigeoka et al., 2019) and *Arabidopsis thaliana* (Luo et al., 2020)] both supportive and dismissive of the importance of specialized translation during early embryogenesis, gametogenesis, fertilization, and neurogenesis. We review these new studies and how they have built upon previous understanding of specialized ribosomes (Guo, 2018). Clearly, there is a great deal of interest in determining whether specialized ribosomes exist and their importance to normal development in a range of systems, rather than just to human disease. In fact, developmental biology seems to be the field in which the functional relevance of ribosome heterogeneity is being most robustly assessed.

## 2 THEORY OF SPECIALIZED RIBOSOMES

**FIGURE 1:**
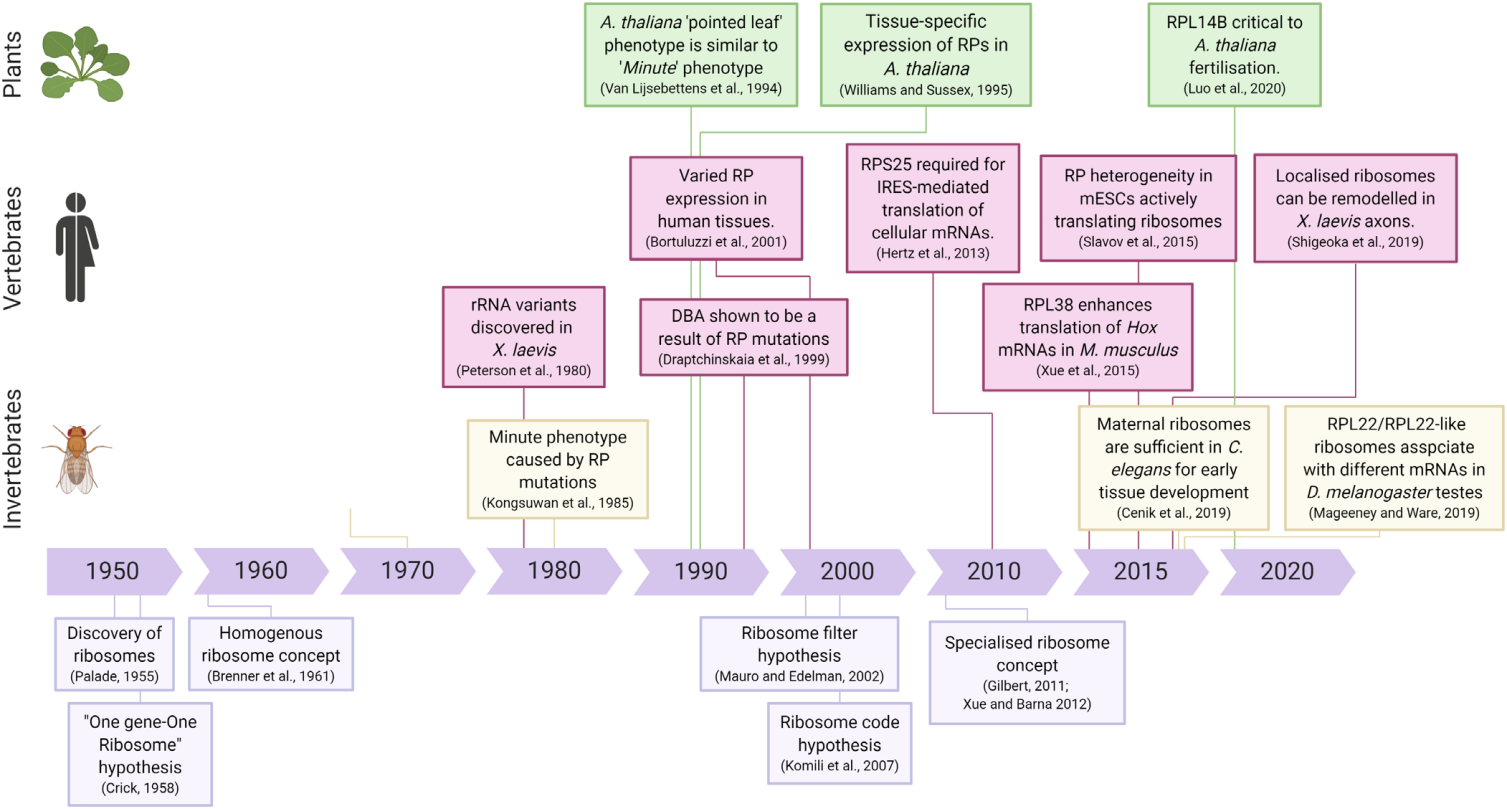
Timeline of our understanding of specialized ribosomes. The theory of specialized ribosomes initially started back in 1950s but in the last 10 years more evidence and examples have been characterized

The theory of ribosome specialization was first suggested in the 1950s when differences in ribosome size and shape were identified (Palade, 1958), and Crick discussed his one gene–one ribosome–one protein hypothesis (Figure 1). However, this concept was not generally accepted and or debated again until 2002 with the emergence of the “Ribosome Filter Hypothesis.” This model suggests that translation might be stimulated or inhibited by the ribosomal subunits regulating how the ribosome interacts with specific mRNAs (Mauro & Edelman, 2002). It was then proposed that a “ribosome code” existed and functioned in a similar manner to the histone code (Komili et al., 2007). It was also hypothesized that a subset of ribosomes are dedicated to the synthesis of antigens for presentation in the human immune system, termed “immunoribosomes” (Yewdell & Nicchitta, 2006). This idea has been further developed since 2011 into the concept of “specialized ribosomes.” Specialized ribosomes are defined as ribosomes that translate specific pools of mRNAs or facilitate translational regulation through unique interactions with ribosome‐associated factors (Genuth & Barna, 2018; Gilbert, 2011; Xue & Barna, 2012).

**FIGURE 2:**
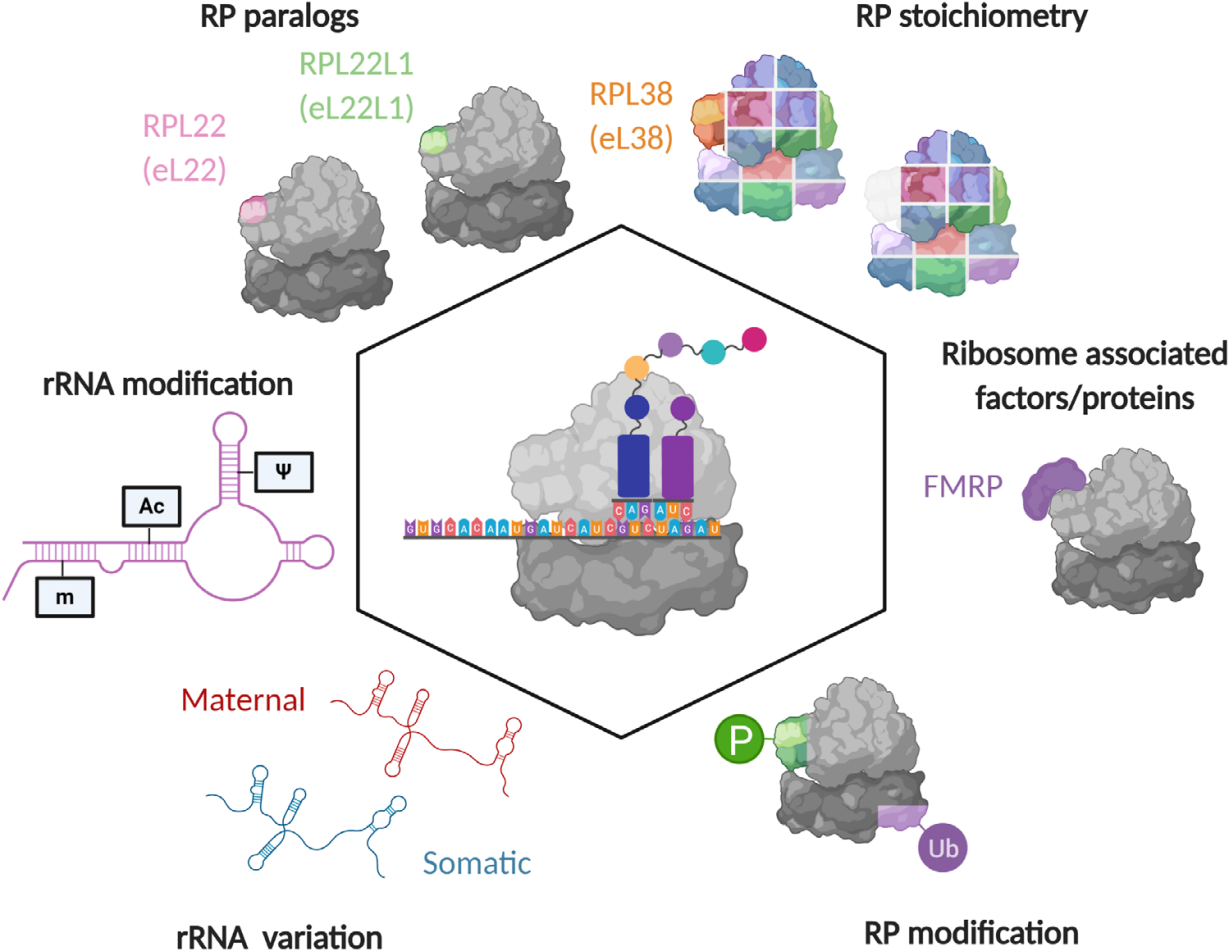
Types of ribosome heterogeneity. Schematic illustrating the six different ways ribosome heterogeneity has been found to occur

The eukaryotic ribosome is highly conserved with both yeast and mammalian ribosomes comprising of 80 RPs and 4 rRNAs. However, there is now compelling evidence from a variety of organisms [mouse embryonic stem cells (Shi et al., 2017), mouse (Hebras et al., 2020), yeast (García‐Marcos et al., 2008), human cell lines (Krogh et al., 2016), fruit flies (Mageeney et al., 2018), zebrafish (Locati, Pagano, Girard, et al., 2017), and plants (Hummel et al., 2015)] that RP and rRNA composition can vary. In fact, six key means of generating ribosome heterogeneity have been characterized (Figure 2): (i) substitution of RP paralogs (Kearse et al., 2011), (ii) differential stoichiometry of RPs (Kondrashov et al., 2011; Shi et al., 2017), (iii) additional protein components (Simsek et al., 2017), (iv) posttranslational modification of RPs (Carroll et al., 2008), (v) rRNA variation (Locati, Pagano, Girard, et al., 2017; Peterson et al., 1980), and (iv) rRNA modifications (Natchiar et al., 2017). All these compositional changes have the potential to form functionally specialized ribosomes.

## 3 POTENTIAL MECHANISMS OF SPECIALIZED RIBOSOMES

**FIGURE 3:**
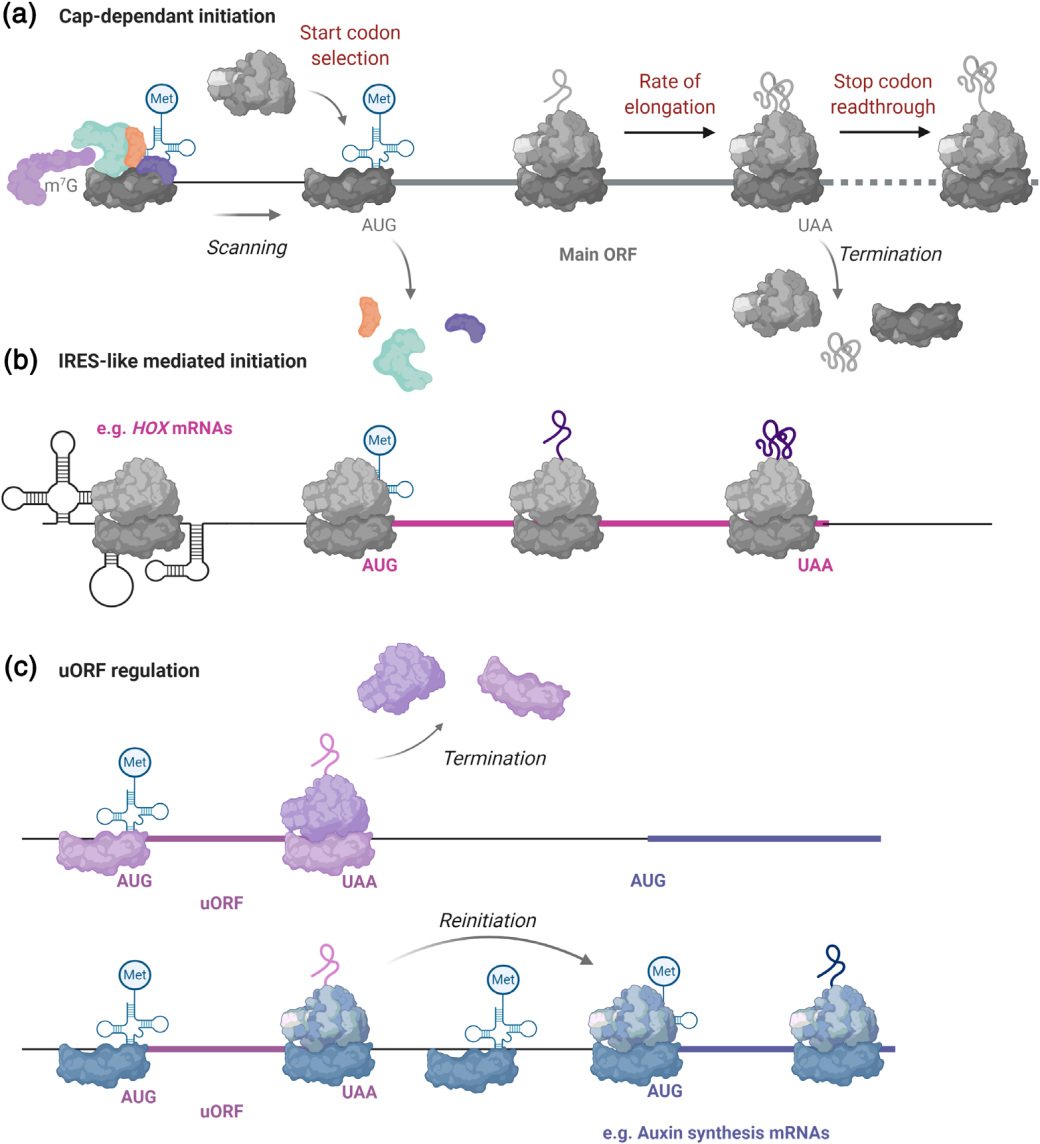
Mechanisms of translation initiation and regulation. Schematic depiction of how specialized ribosomes could mechanistically affect translation

Although substantial evidence exists to support ribosome heterogeneity, the data toward functional specialization are limited and therefore, the concept more controversial. So far, there is limited mechanistic detail to explain how different ribosome populations differentially regulate mRNA translation in the same cells or at a specific developmental time point. Therefore, current efforts are concentrated in elucidating how changes to ribosome composition might enable translation of specific groups of mRNAs. Changes to ribosome composition could potentially modulate translation in a number of ways (Figure 3(a)): (i) recruitment of 40S to 5′‐UTR, (ii) selection of start codon, (iii) rate of elongation, (iv) translation fidelity, and (v) stop‐codon recognition. Examples of only a few of these mechanisms have so far been discovered.

The best characterized mechanism is the specialization produced by RPL38/eL38 in mouse. *HOX* mRNA 5′‐UTRs appear to contain internal ribosome entry site (IRES)‐like elements that help recruit ribosomes via RPL38 (Xue et al., 2015) (Figure 3(b)), and therefore their translation is dependent on RPL38, unlike other mRNAs (Table 1). Specialization by RPL38 is discussed in great detail in Section 4.2 later in the review. Recruitment of ribosomes to 5′‐UTRs has also been found to be dependent on RPS25/eS25 in many cellular IRESes, for example, c‐Myc (Hertz et al., 2013). More indirect evidence points to additional mechanisms for other RPs. For example, RPL3/uL3 contributes to translational fidelity (Al‐Hadid et al., 2016) and so its absence or modification could affect peptide composition. Furthermore, ribosome profiling has revealed 100s of stop‐codon read‐through events, which are subject to regulation in yeast, *Drosophila* and humans (Dunn et al., 2013). These stop‐codon events might provide a point at which specialized ribosomes could regulate termination of translation. The mammalian paralog pair RPL39L/eL39L and RPL39/eL39 share 92% sequence similarity and are located near the peptide tunnel exit of the ribosome (Zhang et al., 2013). Intriguingly, an Arg28Gln difference between them is conserved across mammals (Wong et al., 2014). This variation makes the mRNA tunnel more positively charged and might affect translational velocity, since translating positively charged residues is a limiting factor on elongation speed (Charneski & Hurst, 2013). However, the link between these potential mechanisms and the importance of ribosome heterogeneity to development is currently lacking, but it is a highly active area of research.

**TABLE 1:** Summary of examples of ribosome specialization

Ribosomal component	Organism	Developmental process	mRNAs translated	Phenotype	Mechanism	References
RPL38/eL38	*Mus musculus*	Body patterning and neurogenesis	*HOX* mRNAs	Homeotic transformation	IRES‐like 5′‐UTR element	Kondrashov et al. (2011), Xue et al. (2015)
RPL22‐like/eL22‐like	*Drosophila melanogaster*	Spermatogenesis	Translation mRNAs	Unknown	Unknown	Mageeney et al. (2018), Mageeney and Ware (2019)
RPS5b/uS7b	*D. melanogaster*	Oogenesis	Electron transport chain and metabolic process mRNAs	Female sterility	Unknown	Kong et al. (2019)
RPS25/eS25	Mouse ESC	Stem cells	Vitamin B12 pathway mRNAs	Unknown	Unknown	Shi et al. (2017)
RPL10A/uL1	Mouse ESC	Stem cells	Extracellular matrix organization, glycosphinoglipid metabolic processes	Unknown	IRES‐like 5′‐UTR element	Shi et al. (2017)
RPS4X/eS4X	*Xenopus laevis*	Neurogenesis	Ribosomal protein (RP) mRNAs	Reduced axon branching	Loop motif in 5′‐UTR	Shigeoka et al. (2019)
18S rRNA (maternal vs. zygotic)	*Drosophila rerio*	Embryogenesis	Maternal mRNAs	Unknown	5′‐UTR element	Locati, Pagano, Ensink, et al. (2017), Locati, Pagano, Girard, et al. (2017)
RPL24B/eL24B	*Arabidopsis thaliana*	Gametogenesis	*ARF3* mRNA, *ETT* mRNA, *MP* mRNA	Female infertility	uORF	Rosado et al. (2012)

*Note*: Specific examples of ribosome specialization, across different organisms and various developmental processes. Key features of specialization are indicated including: which mRNAs are translationally regulated, phenotype and mechanistic insight.

## 4 EVIDENCE FOR RIBOSOME SPECIALIZATION

Two significant factors have contributed to the logic behind the idea of “specialized ribosomes”: (i) prevalence of tissue‐specific RP expression of particular RPs, and (ii) distinctive phenotypes when specific RP genes are disrupted (Dinman, 2016). Both these factors are underpinned by the potential importance of ribosome specialization during development, giving rise to phenotypes at different points and generation of specialized tissues during development.

### 4.1 Tissue‐specific expression

Many organisms possess paralogs of RPs including flies, humans, mice, and plants. Transcriptomic and proteomic data indicate that many of these paralogs are expressed in a tissue‐specific manner. For example, RPL10L/uL16L and RPL39L/eL39L exhibit testis‐specific expression in rodents (Sugihara et al., 2010). Presumably, one of the RP paralogs is present in each ribosome but different cells might express different paralogs that are incorporated into the ribosome. Paralogs are evolved from a common ancestor within an organism and are therefore likely to have similar functions, but functions may have diverged over time. The numbers of RPs paralogs vary tremendously between different organisms, as well as the amino acid sequence identity shared between a pair or group of paralogs. Although paralogs may be expressed differentially, not all paralogs are likely to be functionally distinct. Paralog‐specific requirements, like those seen for *ASH1* mRNA in *Saccharomyces cerevisiae* suggest that paralogs can be functionally different, contribute to ability to regulate ribosome structure and therefore function as a result of differences in their amino acid sequence (Komili et al., 2007). Various plants [e.g., *Oryza sativa* (rice) and *Arabidopsis*] have also been found to have tissue‐specific expression of RPs. For example, there is specific expression of RPS5A/uS7A, RPS18A/uS13A and RPS16B/uS9B paralogs in apical and root meristems while other paralogs within the family are expressed in specific nonproliferating tissues (Van Lijsebettens et al., 1994; Weijers et al., 2001; Williams & Sussex, 1995). The expression and function of RP paralogs in plants will be discussed in more detail later.

### 4.2 Distinctive phenotypes

Although one might presume that all RPs are required for all translation events, several lines of evidence suggest this is not the case. In budding yeast, bud‐site selection is dependent on RPL7A/uL30A, RPL22A/eL22A, RPL12A/uL11A, and RPS18B/uS13B but not their paralogs. These specific RPs are required for the translation of *ASH1* mRNA which is essential to bud‐site selection (Komili et al., 2007). This suggests that these proteins exert a function that is required for the translation of this specific mRNA, which cannot be performed by their paralogs. A similar dependency on specific RPs is also seen during viral infection. The vesicular stomatitis virus mRNA is highly sensitive to RPL40/eL40 depletion, more so than other RPs (Lee et al., 2013). Many viruses use IRES to initiate translation of their mRNAs during host cell translation shut‐off (Figure 3(b)). These IRESes can be more dependent on some specific RPs than others. For example, HCV IRES translation requires RPS25/eS25 (Landry et al., 2009).

It has long been appreciated from *D. melanogaster* that mutations in RP genes result in specific phenotypes. Null mutants for RP genes are embryonic lethal, as one might expect, while haploinsufficient *D. melanogaster* RP mutants have various phenotypes that are dominant. Some phenotypes are common between RP mutants while others are distinct. The *Minute* phenotype, originally characterized by Calvin Bridges as short and narrow thoracic bristles (Bridges & Morgan, 1923), was found to be the result of mutations in RP genes (e.g., *RPS5A/uS7A*, *RPL38/eL38*, *RPS10B/eS10B*) (Marygold et al., 2007; Kongsuwan et al., 1985) (Figure 1). Other phenotypes that *Drosophila* RP mutants exhibit include prolonged larval development, reduced body size, and infertility (Lambertsson, 1998). *Minute*‐like phenotypes have also been characterized in plants including *A. thaliana*, with dwarfed stature, delayed development, and abnormal leaf morphology (Van Lijsebettens et al., 1994; Williams & Sussex, 1995) (Figure 5). The caveat with specific phenotypes from RP mutants is that they are not necessarily the result of a requirement for an RP in the translation of specific mRNAs, that is, ribosome specialization. Phenotypes could be the result of reduced levels of general mRNA translation or nonribosomal functions of these proteins.

One of the most significant examples of RP phenotypes that is translation dependent and therefore provides substantial evidence for ribosome specialization is RPL38/eL38 in mice. RPL38 mutant mice exhibit impaired neural specification and a homeotic transformation phenotype (Kondrashov et al., 2011). These highly specific phenotypes only result when RPL38 is lacking, not when other RPs were disrupted. The homeotic transformation phenotype is the result of an inability to translate *HOX* mRNAs. Therefore, the presence of RPL38 in the ribosome is essential to the translation of *HOX* mRNAs, while presumably general translation can proceed to enable the mouse to survive without RPL38 (Kondrashov et al., 2011). This is achieved by the recruitment of ribosomes to HOX 5′‐UTRs via IRES‐like elements and RPL38. In the absence of RPL38, ribosomes are able to translate other mRNAs but not *HOX* mRNAs (Xue, ; Xue et al., 2015).

**Insights into ribosome specialization from human diseases**

Mutations in human ribosome genes have also been identified that contribute to a group of human diseases termed ribosomopathies. They are caused either by mutations in RPs or proteins required for ribosome biogenesis. The most well characterized is Diamond Blackfan anemia (DBA), which results from haploinsufficiencies in several different RP genes (*RPS24/eS24*, *RPS17/eS17*, *RPL35A/eL33*, *RPL5/uL18*, *RPL11/uL5*, *RPS7/eS7*, *RPL36/eL36*, *RPS15/uS19*, *RPS27A/eS31*) and *RPS19/eS19*, which is mutated in 25% of patients (Narla & Ebert, 2010). DBA is primarily a bone marrow disorder but patients also exhibit a range of physical abnormalities. Given that ribosomes are required in all cells the variety of specific clinical symptoms suggests that different cells and tissues have differing sensitivities to the levels of various RPs. Reticulocytes, for example, seem particularly sensitive to such imbalances. It is worth noting that dysfunction of ribosome biogenesis can trigger p53 activation. RPL5/uL18, RPL11/uL5, and RPL23/uL14 mutations have each been found to result in stabilization of p53 and therefore cell‐cycle arrest and apoptosis. Therefore some of the symptoms exhibited in ribosomopathies may be dosage dependent rather than resulting from specialized ribosomes.

Recently, a link has been discovered between rRNA modifications and cancer, leading to the theory of oncoribosomes. These could be specialized during cancer to drive high levels of translation and uncontrolled cell division, by leading to increased levels of RPs through translational regulation. In fact 45% of colon cancers exhibit loss of the specific rRNA modification m^1^acp^3^Ψ (Babaian et al., 2020).

## 5 GAMETE DEVELOPMENT AND GAMETOGENESIS

The importance of translational regulation during gametogenesis is well established in both testes (Kleene, 2003) and ovaries (Lasko, 2012). This is exemplified by the involvement of gonad‐specific eukaryotic initiation factors (eIFs) that are present in numerous species [e.g., *D. melanogaster*: eIF4E‐3 and eIF4G2 in spermatogenesis (Ghosh & Lasko, 2015; Hernández et al., 2012), *C. elegans*: IFE‐1 in oogenesis and spermatogenesis (Henderson et al., 2009)]. These specialist eIFs are required for the expression of proteins that are integral to several developmental processes in gametogenesis (e.g., postmeiotic differentiation) and could mediate the translation of mRNA transcripts selectively. RP haploinsufficiency (e.g., *D. melanogaster*: *Minute* phenotype) and null mutations [e.g., RPL29/eL29 in mice (Aravindan et al., 2014)] can lead to impaired fertility. This raises the possibility that heterogeneous ribosomes with specialized function contribute an extra layer of regulation during sex cell development.

### 5.1 Spermatogenesis

**FIGURE 4:**
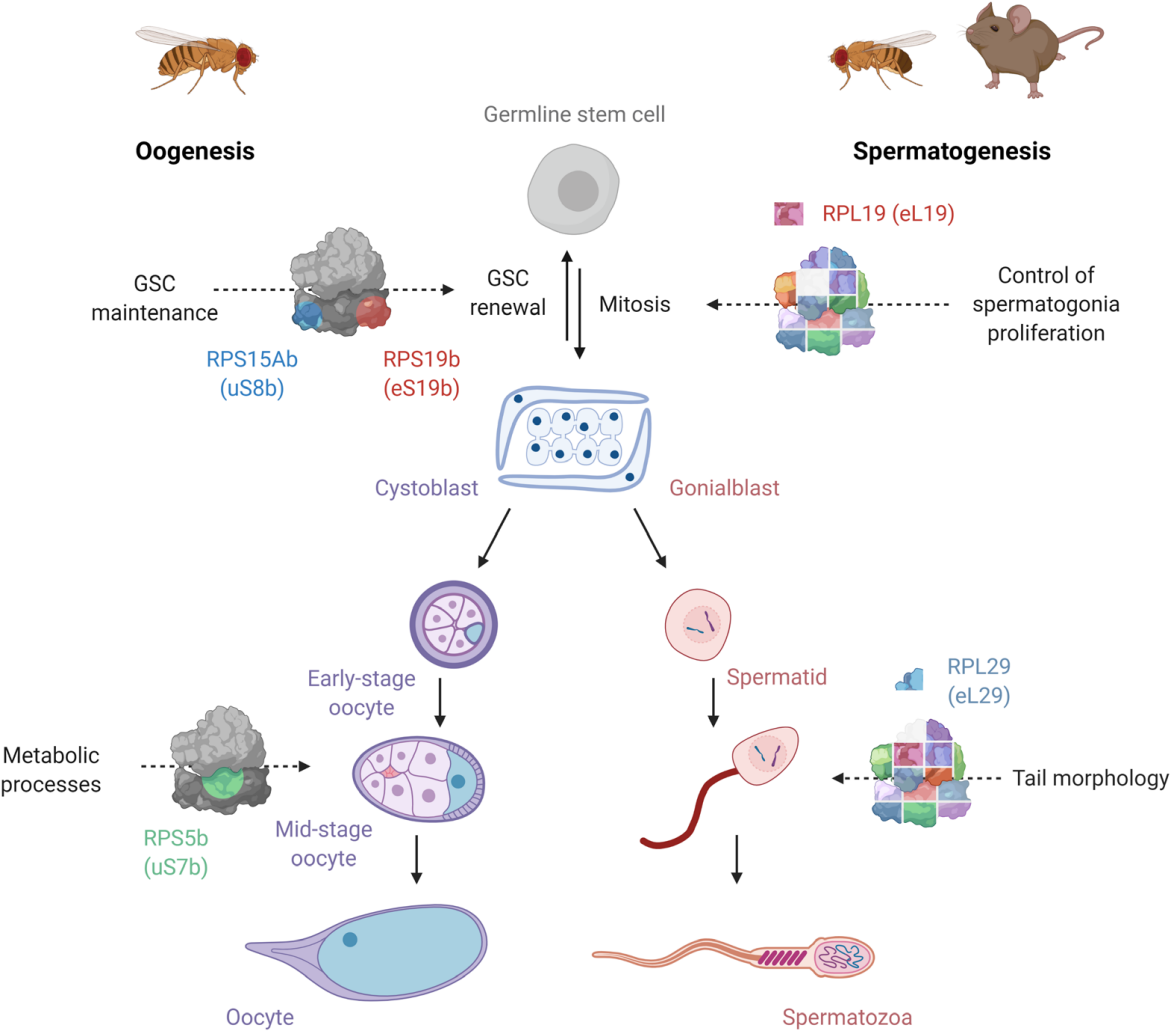
Role of ribosome heterogeneity in gametogenesis. Schematic diagram highlighting the potential roles of ribosome heterogeneity in oocyte development (*Drosophila melanogaster*) and sperm cell development (*D. melanogaster*, *Mus musculus*) from studies using different model organisms

Several lines of evidence suggest that specialized ribosomes function during spermatogenesis. Male infertility can arise from the loss or altered expression of RPs, with specific abnormalities in sperm cell development depending on the RP affected. In *Drosophila*, cell‐specific RPL19/eL19 depletion in the germline stem cells (GSCs) leads to the loss of the stem cell niche, while knockdown during the mitotic phase results in the overproliferation of spermatogonia (Yu et al., 2016). Spermatozoa from *rpl29/eL29* null mice exhibit abnormal flagella (i.e., “dag” defects) that renders them immobile (Figure 4). Gene expression analysis in humans has also linked the upregulation and downregulation of several RPs to reduced sperm mobility and idiopathic infertility (Bansal et al., 2015). These observations suggest that several RPs play specific and distinct roles during different stages of spermatogenesis. However, it is not yet clear whether the differential incorporation of RPs alters the translational priorities of the ribosome during spermatogenesis.

Several RPs have evolved paralogs that exhibit testis‐specific expression in insects and mammals indicating a potential mechanism of ribosome specialization during spermatogenesis. In *D. melanogaster* RpL22‐like/eL22‐like has been identified as tissue restricted with the highest levels in the adult male germline (Mageeney et al., 2018) and, four paralogs are specifically expressed in the testis of mammals [RPS4Y2/eS4Y2, RPL22L1/eL22L1, RPL39L/eL39L and RPL10L/uL16L (Lopes et al., 2010; Nadano et al., 2002; Sugihara et al., 2010)]. Why such diverse groups of testis‐specific paralogs have evolved across animals is unclear but may be explained by meiotic sex chromosome inactivation (MSCI), which is exclusive to males. During the late meiotic stages of sperm cell development, the sex chromosomes become transcriptionally silent and their autosomal paralogs are expressed as a compensatory mechanism (Turner, 2015). Given that most testis‐specific paralogs are encoded on autosomes and have a sex‐linked canonical counterpart (with the exception of RPS4Y and RPL22 in mammals), this could explain the testis‐specific expression of paralogous RPs and argue against specialization. While evidence for this exists for human RPL10 and RPL10L (Jiang et al., 2017), the other paralogs have yet to be investigated. On the other hand, paralog expression driven by MSCI could provide the ideal context for neofunctionalization, which could give rise to specialized ribosomes.

Interestingly, both RPL22‐like/eL22‐like (*Drosophila*) and RPL22L1/eL22L1 (humans) are testis specific. In fact, ribosomes containing either RPL22 or RPL22‐like in *Drosophila* testes were recently shown to preferentially translate different sets of mRNA (Mageeney & Ware, 2019). RPL22 ribosomes were found to associate with mRNAs involved in organ development, while RPL22‐like ribosomes preferentially translated mRNAs involved in transport and translation (Table 1). RPL22 ribosomes did show a preference for several mRNAs with functions during spermatogenesis, however, the importance of RPL22‐like in spermatogenesis remains unclear. In *D. melanogaster*, ubiquitous knockdown of either RPL22 paralog is embryonic lethal (Mageeney et al., 2018). While conditional knockout of RPL22‐like during early larval development had a marginal effect on testis development, early spermatogonia targeted RNAi (via Bam‐gal4) in the adult did not, suggesting RpL22‐like is not required for sperm cell development (Mageeney et al., 2018). Rescue experiments do suggest that the function of these two paralogs is functionally distinct. To determine if RPL22 and RPL22‐like are expressed in a cell type‐specific manner within the testis, which might indicate importance to spermatogenesis, we have analyzed published single‐cell RNA sequencing data from *D. melanogaster* testes (Witt et al., 2019). This indicates that RPL22‐like is highly expressed in GSCs and early spermatogonia before subsequently declining as development progresses (Witt et al., 2019). While RPL22‐like protein has been detected throughout sperm cell development, it seems possible that RPL22‐like may be important in particular processes within GSCs and early spermatogonia, such as maintaining the male germline niche via asymmetric GSC division or de‐differentiation of early spermatogonia, given its high expression in these cell types (Herrera & Bach, 2018). GSCs are lost as male flies’ age and therefore, the processes that replenish them are important to maintain male fertility. This could explain the lack of phenotypes observed from experiments that often use young and infrequently mated male flies. Nonetheless, it is currently unclear whether RPL22 paralogs are specialized during spermatogenesis. In zebrafish, both RPL22 paralogs exhibit extra‐ribosomal functions in the nucleus that are critical to development (Zhang et al., 2013; Zhang et al., 2017), and so, the testis‐expression of RPL22‐like in flies and humans might also include extra ribosomal function(s).

### 5.2 Oogenesis

As in the testis, there is evidence to indicate that specialized ribosomes may exist and function in the ovary. From a human disease perspective, the altered expression of specific RPs has been linked to polycystic ovary syndrome (Ramly et al., 2019), suggesting potential roles in ovarian development. Translational control is essential in the ovary and has been extensively characterized (Richter & Lasko, 2011). Whether specialized ribosomes add to this complex regulation is unknown, but data from studies in model organisms, such as *D. melanogaster* and *Drosophila rerio*, indicate that specialized ribosomes could play integral roles during oocyte development. *Drosophila* RNAi screens have revealed that paralogous RPs play distinctive roles at different stages of oocyte development. These include GSC maintenance, for example, RPS15Ab/uS8b and RPS19b/eS19b (Sanchez et al., 2016; Yu et al., 2016) and early differentiation in the ovary germarium, for example, RPS19a/eS19a (Sanchez et al., 2016) (Figure 4). This suggests that the ribosomes they are part of translate distinct mRNA pools.

The RPS5a/uS7a and RPS5b/uS7b paralog pairs have been profiled in detail in *D. melanogaster* and may have distinct roles in oocyte development. RPS5a is expressed in the follicle cells surrounding the germline while RPS5b is present in the germline cells. Following mitosis, the developing egg slows its growth and undergoes a nutritional check prior to synthesizing yolk proteins (Raikhel & Dhadialla, 1992). The check monitors whether the immature oocyte can complete development and, if there are inadequate nutrients or resources to maintain protein synthesis, the developing oocyte undergoes apoptosis (Terashima & Bownes, 2005). *RPS5b* mutants induce apoptosis at the same stage of oocyte development where the nutritional check occurs, resulting in sterile females (Figure 4). In contrast, *RPS5a* RNAi is embryonic lethal. Although there is no evidence that RPS5b is involved in this checkpoint, RPS5b could have an indirect effect by preferentially translating proteins that help to sustain cellular resources required to continue development (Kong et al., 2019). Since RPS5 is located near the mRNA channel, it may participate in mRNA selection and the recruitment of ribosomes onto mRNAs. RIP‐Seq of RPS5a and RPS5b supports this idea, since RPS5b preferentially associates with mRNAs involved in mitochondrial and metabolic functions (e.g., electron transport chain) (Kong et al., 2019). While these observations indicate that RPS5b incorporation may confer ribosome specialization, the fertility defect in *RPS5b* mutants can be rescued by RPS5a overexpression, suggesting that the phenotype may be a consequence of altered RPS5 levels rather than translational control by RPS5b specifically. Thus, although it has been established that RPS5a and RPS5b containing ribosomes may translate different mRNAs, this may not be essential to oocyte development (Table 1).

## 6 EMBRYOGENESIS

Newly fertilized oocytes are transcriptionally silent and rely on maternally inherited mRNAs and cellular organelles. This includes a pool of ribosomes that drives protein synthesis before the maternal to zygotic transition (i.e., when de novo ribosome synthesis begins). Conceptually, this might represent an ideal time for the embryo to employ specialized ribosomes to regulate translation. Heterogeneity in the embryo has been so far characterized at the level of rRNA, as well as RP composition.

Eukaryotes have 50–5000 copies of each rRNA gene in their genomes, with humans having ~400 copies. Differences in which exact rRNA genes are expressed could facilitate the generation of different ribosome populations. Expression of these rRNA genes can be affected by environmental factors during early development. For example nutritional status regulates epigenetic marks during pregnancy in mice, which regulates expression of different rRNA genes (Holland et al., 2016). Distinct rRNA genes (for each 5S, 5.8S, 18S, and 28S rRNA) are expressed in the developing zebrafish oocyte, which generate distinct rRNA variants at the sequence level (Locati, Pagano, Girard, et al., 2017). Once fertilized, the maternal type variants are steadily replaced by somatic versions following gastrulation. This pattern of expression may suggest that the ribosome plays dynamic roles during the early and later stages of embryonic development. Supporting this idea, in silico analysis suggests that the maternal type 18S rRNA allows initiating 40S subunits to preferentially interact with the 5′‐UTRs of maternally deposited mRNAs (Locati, Pagano, Girard, et al., 2017). While the functional implications of this finding require further investigation, this phenomenon could serve to “filter” paternally inherited mRNAs and reduce the likelihood of ribosome recruitment on sperm mRNAs (Mauro & Edelman, 2002; Zhao et al., 2006) (Table 1).

In contrast to the potential specialization of ribosomes through rRNA, it has been shown that maternally inherited ribosomes are sufficient to complete embryogenesis and early tissue development in *C. elegans* (Cenik et al., 2019). By depleting rRNA genes and introducing loss‐of‐function mutations in five RP proteins, it was found that newly synthesized ribosomes are not required for differentiation of cells. This suggests that de novo synthesis of potentially specialized ribosomes is unnecessary in *C. elegans*, yet this does not rule out the possibility of existing ribosomes being remodeled in order to alter embryonic translational regulation (see Section 7). Nonetheless, homozygous null or haploinsufficient mutations in other animals can lead to embryonic lethality or impaired tissue development, suggesting that ribosomes with the capacity to regulate translation could be important during early embryogenesis.

Unsurprisingly, translational control is critical to proper embryonic development (Tahmasebi et al., 2018). Repression of cap‐dependent translation initiation is a key feature of embryonic stem cells. Translation of some pluripotency factors (e.g., Nanog, c‐Myc) continues via cap‐independent mechanisms, allowing cells to maintain an undifferentiated state (Friend et al., 2015; Ingolia et al., 2011). To circumvent the inhibition of translation initiation, specialized ribosomes could be recruited to mRNAs directly via 5′‐UTR IRES‐like elements and thus, allow the synthesis of proteins that are integral to embryonic development. Evidence supporting this theory comes from analysis of ribosomal populations in mouse embryonic stem cells, showing that only a proportion of ribosomes contain the core RPs RPL10A/uL1 and RPS25/eS25 (Shi et al., 2017). Each of these RPs is required for the translation of distinct mRNAs involved in the cell cycle and the synthesis of cell surface lipids that are important for tissue development during embryogenesis (Shi & Barna, 2015; Yamashita et al., 1999) (Table 1). These specialized ribosomes could preferentially target IRES‐like elements in the 5′‐UTRs of specific mRNAs. Both RPL10A and RPS25 are able to interact with viral IRESes (Landry et al., 2009; Shi et al., 2017). RPS25/eS25 binds to and is also required for the translation of several cellular IRESs including embryonic factors (e.g., c‐Myc) (Hertz et al., 2013).

Several other types of ribosome specialization have been characterized in ESCs. For example, protein factors have been identified that associate with translating ribosomes and contribute to translational regulation (Simsek et al., 2017). PKM2 binds elongating ribosomes and is specifically associated with a group of mRNAs translated at the ER and regulates their translation (Simsek et al., 2017). RP modifications have also been discovered in mouse ESCs, although their functions are poorly understood. Ubiquitin fold modifier (UFMylation) was discovered on numerous sites across different RPs (Simsek et al., 2017). Further work will be required to explore whether such modifications contribute functionally to embryo development.

## 7 NEURAL DEVELOPMENT

Neurons, with their distinct dendritic and axonal appendages that often extend far beyond the cell body, are highly dependent on localized translation (Hafner et al., 2019). For instance, in developing axons, the cytoskeleton in the leading growth cone is constantly remodeled by localized translation in response to guidance cues (e.g., Netrin‐1) that help navigate the appendage to where the axon may branch out and form synaptic complexes [reviewed previously (Boyer & Gupton, 2018)]. Thus, regulation of protein synthesis in a spatial and temporal manner allows the rapid adjustment of the local proteome in response to local stimuli. Subsequently, a local pool of ribosomes is essential to establishing and maintaining the neurocircuitry. Specialized ribosomes have been suggested to play an important part of this local translation regulation.

Compositional changes that drive ribosome specialization may arise in a localized manner during neurodevelopment. Analysis of the axonal transcriptome has identified thousands of localized mRNAs that are pivotal to synapse development, including a subset of RP transcripts and ribosome assembly factors (Briese et al., 2016; Shigeoka et al., 2019; Zivraj et al., 2010). This striking observation led to the discovery that axonal ribosomes can be remodeled through the exchange of RPs present on the ribosomes outer surface for RPs that are locally synthesized (Shigeoka et al., 2019). Not only is this in stark contrast to the widely held view that ribosomes are static in composition once assembled, it also demonstrates that the modulation of ribosome composition can take place in situ and does not have to occur in the nucleolus during ribosome biogenesis. Localized compositional changes could give rise to specialized ribosomes, which target and translate mRNA critical to synapse function and maintenance, or simply repair damaged ribosomes.

Mechanistic insight into the potential for localized specialized ribosomes in neurons comes from recent ribosome profiling of rat hippocampal neuropils. Specific mRNAs were found to be differentially distributed between monosomes and polysomes (Biever et al., 2020). Given that monosomes and polysomes engage in different levels of mRNA translation, ribosome specialization could regulate protein synthesis by changing an mRNA's association with monosomes and polysomes. In fact, in mouse embryonic stem cells, RPS4X/eS4X is stoichiometrically enriched in monosomes. Thus, in the axonal context, it could help to promote the translation of mRNAs that are required during axon development (Slavov et al., 2015). In support of this idea, knockdown of locally synthesized RPS4X reduces rates of axonal translation. This impedes synaptic development by reducing the number and complexity of axonal branches (Shigeoka et al., 2019) (Table 1). Together, these findings suggest that RPS4X could mediate translational regulation of distinct mRNAs directly, or by influencing their distribution across monosomes and polysomes. Another example of potential neuronal specialization is exemplified by the transmembrane receptor, deleted in colorectal cancer (DCC). DCC facilitates translation initiation in response to Netrin‐1 extracellular signaling (Tcherkezian et al., 2010). This activity is dependent on specific RPs (e.g., RPL5/uL18) and is postulated to increase the number of actively translating polysomes.

Several ribosomopathies display severe neurodevelopmental defects, which may result from the disruption of specialized ribosome populations. Common disease characteristics, such as microcephaly, are the result of decreased neuronal progenitor cell (NPC) proliferation or NPC death (reviewed in Hetman & Slomnicki, 2019). These likely result from reduced cellular translational capacity, since high translation rates are required to sustain NPC proliferation during development. For instance, a loss of function mutation in RPL10/uL16 causes microcephaly and severely impaired cognitive function (Brooks et al., 2014). Intriguingly, however, specific RPL10 mutations (Leu206Met, His213Gln) have been linked to autism spectral disorders (Chiocchetti et al., 2011; Klauck et al., 2006) and could be the result of disruptions to specialized ribosome populations. Previously characterized cancer‐causing mutations in RPL10 affect IRES‐mediated translation of BCL‐2 mRNA, but not global translation (Kampen et al., 2019). Therefore, dependency of specific RPs seems a common feature of cellular IRESes. BCL‐2 is expressed at lower levels in autism (Fatemi et al., 2001), although we do not know how the Leu206Met and His213Gln mutations may affect BCL‐2 IRES‐mediated translation, this could provide a potential explanation for how RPL10‐specialized ribosomes are linked to autism.

Further insight into the potential importance of neuronal ribosome heterogeneity comes from Fragile X Syndrome (FXS). FXS is an inherited disorder caused by decreased expression of the fragile X mental retardation protein (FMRP) (Hagerman et al., 2017). FMRP is an RBP important for synaptic development and plasticity and regulates the translation of numerous mRNAs involved in cytoskeleton organization, membrane trafficking, and synaptic signaling. Recent evidence suggests that FMRP may play a role in generating specialized ribosomes. FMRP has been shown to regulate dynamic 18S and 28S rRNA 2′O‐Me modifications by associating with C/D box small nucleolar RNAs within the nucleus (D'Souza et al., 2018). Thus, FMRP can contribute to neuronal ribosome heterogeneity through rRNA diversity. As a ribosome‐associated factor, FMRP may also alter the ribosome's preference for specific mRNAs directly. It has been long known that FMRP associates with actively translating polysomes and while this could give rise to specialized ribosomes (Stefani et al., 2004), the function behind this association and its implications in neurodevelopment remains unknown (Goering et al., 2020).

Heterogeneity of rRNAs may also have roles in neuronal development. rRNA levels have been found to vary during neuronal development and across different tissues in mice. Distinct patterns of rRNA modifications have also been characterized in mice and zebrafish and seem to be most distinct during brain development. However, these variations in ribosome composition seem to have little functional effect on translation (Hebras et al., 2020). Disruptions to rRNA biogenesis do result in striking neuronal phenotypes, such as reduced differentiation, but this is likely due to an overall reduction in rRNA levels and induction of p53‐dependent apoptosis (Bouffard et al., 2018).

## 8 PLANTS

### 8.1 Ribosome heterogeneity in plant ribosomes

Although the gross architecture of ‘the plant ribosome’ is comparable to that of the animal kingdom (80 RPs, 4 rRNAs) (Barakat et al., 2001), the genetic diversity encoding plant RPs far surpasses its animal counterparts. This vast genomic complexity resulted from the selective retention of ribosomal genes following extensive genomic duplications in multiple plant lineages (Blanc & Wolfe, 2004; Thomas et al., 2006). All angiosperms (flowering plants) including *A. thaliana* are likely descended from ancestors that underwent at least two whole genome duplications (Jiao et al., 2011; Vision et al., 2000; Wang et al., 2011). The diploid *A. thaliana* has 242 annotated RP‐encoding genes (Hummel et al., 2015), with between 2 and 7 paralogs in each gene family (Barakat et al., 2001; Hummel et al., 2015). The *Brassica napus* (rapeseed) genome has undergone such extensive genomic expansion (Chalhoub et al., 2014) that it now possesses 996 putative RP‐encoding genes (Whittle & Krochko, 2009); approximately 10 times more than the human genome (Guimaraes & Zavolan, 2016).

Out of the 81 RP‐encoding gene families in *A. thaliana*, only 10 are completely lacking in sequence divergence in protein‐coding regions (Carroll et al., 2008), raising questions regarding the functional equivalency of the remaining 71 RP families. While only 10 *A. thaliana* RP genes have been annotated as pseudogenes, 165 encode proteins found in cytosolic ribosomes, suggesting that the majority function as components of the translational machinery (Hummel et al., 2015; Yamada et al., 2003).

Such extensive genomic expansion has yielded unquestionable ribosomal heterogeneity within the plant kingdom (Carroll et al., 2008; Chang et al., 2005; Giavalisco et al., 2005; Hummel et al., 2012; Hummel et al., 2015), providing a breeding ground for functional diversification events (Falcone Ferreyra et al., 2013). Conversely, the retention of multiple RP genes could simply enable (i) sufficient expression of RP transcripts in highly translationally active cells (dosage sensitivity) (Devis et al., 2015; Fujikura et al., 2009; Thomas et al., 2006; Weijers et al., 2001); (ii) the correct balance of components in a molecular pathway [the Gene Dosage Balance Hypothesis (GDBH)] (Casanova‐Sáez et al., 2014; Devis et al., 2015; Rosado & Raikhel, 2010; Thomas et al., 2006); and/or (iii) increased species fitness through redundancy in the core translational machinery.

### 8.2 RP mutant phenotypes hint at ribosome specialization in plants

**FIGURE 5:**
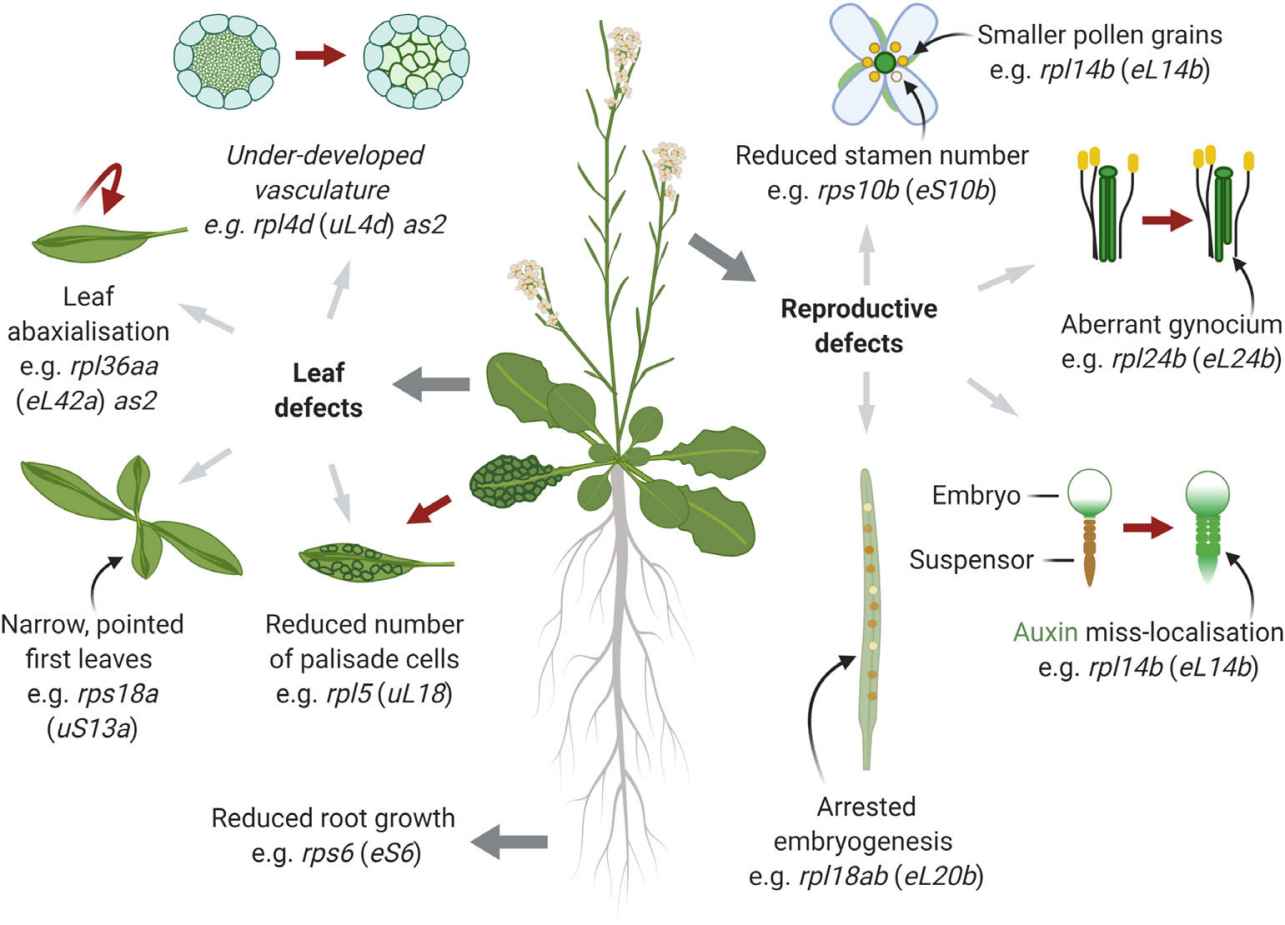
Significant phenotypes of *Arabidopsis thaliana* ribosomal protein (RP) mutants. Defects in leaf morphology are common, including reduced tissue complexity (vasculature and photosynthetic cells) and polarity defects in the *asymmetric leaves1* (*as1*) and *as2* backgrounds (abaxialization; where the upper side of the leaf has the morphology of the underside of the leaf). Aberrant cell fate specification has also been observed in the female gametophyte (gynocium) and during development of the embryo (suspensor)

In plants, RP mutants often display developmental delays and abnormalities that are analogous to the *Minute* phenotype characterized in *D. melanogaster* (described above). Mutants are often smaller, exhibit delayed flowering and display pointed/serrated leaves with simplified/aberrant vasculature and polarity defects (Figure 5) (Fujikura et al., 2009; Horiguchi et al., 2011; Ito et al., 2000; Van Lijsebettens et al., 1994). Within this general phenotype, subtle phenotypic variations have been identified. In leaves, both the rate of cell division and the strength of polarity defects are variable and the intensity of the two is not necessarily positively correlated (Horiguchi et al., 2011). This suggests that RPs have subtly different roles in leaf development.

As in other organisms, some RP mutants display interesting phenotypes in the gametes (Figure 5 and Table 2), for example *rps10b* (*eS10b*) and *rps5a/+* (*uS7a*) display reduced stamen number (Stirnberg et al., 2012; Weijers et al., 2001). *rpl14b/+* (*eL14b*) gametes undergo mitosis and germinate normally, but the pollen grains are smaller and the pollen tube grows more slowly than wild type, resulting in reduced competitiveness (Figure 5). The female gamete displays a reduction in synergid cells, cells that are responsible for guiding the pollen tube to the ovule (Luo et al., 2020). *rpl18ab/+* (*eL20b*) also display reduced pollen tube growth (Yan et al., 2016) and show embryo defects consistent with improper cell fate specification (Xie et al., 2018) (Figure 5). The gynoecium (female reproductive organ) of *rpl24b/eL24b* also displays aberrant development (Nishimura et al., 2005) (Figure 5 and Table 1).

**TABLE 2:** Developmental phenotypes of ribosomal protein (RP) families in *Arabidopsis thaliana*

RP family	RP family size	Amino acid identity %	Mutant developmental phenotype	References
RPS5/uS7	2	94	*rps5a/+*: reduced gamete viability, reduced stamen number, delayed development, aberrant cotyledon vasculature. Phenotypes occur in tissues where expression of 5A and 5B do not overlap *rps5a*: embryo lethal	Weijers et al. (2001)
RPS6/eS6	2	94	*rps6a or rps6b*: slow growth, pointed first leaf, reduced root growth *rps6a/+*, *rps6b/+*: slow growth, pointed first leaf, reduced root growth *rps6a*, *rps6b*: embryo lethal	Creff et al. (2010), Horiguchi et al. (2011)
RPS10/eS10	3	74–78	*rps10b*: reduced stamen number, shoot and floral meristem failure, leaf polarity defects, aberrant auxin responses	Stirnberg et al. (2012)
RPS13/uS15	2	99	*rps13a*: pointed first leaf, aberrant trichome morphology, reduced root growth, late flowering, reduced number of palisade cells.	Ito et al. (2000)
RPS18/uS13	3	100	*rps18a*: narrow, pointed first leaves, reduced growth	Van Lijsebettens et al. (1994)
RPL4/uL4	2	95	*rpl4a* or *rpl4d*: leaf abaxialization and altered vasculature in *as1* background; vacuolar trafficking defects and aberrant auxin responses *rpl4a*, *rpl4d*: embryo lethal; two functional copies required	Horiguchi et al. (2011), Rosado et al. (2012), Rosado et al. (2010)
RPL5/uL18	2	98	*rpl5a*: cotyledon number varies from 1 to 4, leaf polarity defects, needle‐like leave, altered leaf patterning via HD‐ZIPIII‐KANADI pathway *rpl5a or rpl5b*: reduced number of palisade mesophyll cells, narrow leaves, leaf patterning defects	Fujikura et al. (2009), Pinon et al. (2008), Yao et al. (2008)
RPL9/uL6	3	100	*rpl9c*: altered leaf patterning via HD‐ZIPIII‐KANADI pathway Additive effects‐delayed growth early in development, pointed/serrated leaves, delayed flowering *rpl9c* and *rpl9d*: embryo lethal	Devis et al. (2015), Pinon et al. (2008)
RPL10a/uL1	3	91‐96	*rpl10ab*: altered leaf patterning via HD‐ZIPIII‐KANADI pathway	Horiguchi et al. (2011), Pinon et al. (2008)
RPL10/uL16	3	95	*rpl10a/+*: female reproductive defects *rpl10a*: lethal (female gametophytic defects) *rpl10b*: abnormal growth *rpl10c*: wild type	Falcone Ferreyra et al. (2013), Ferreyra et al. (2010), Imai et al. (2008)
RPL14/eL14	2	93	*rpl14b/+*: female reproductive; pollen tube guidance; smaller pollen grains aberrant auxin responses *rpl14b*: embryo lethal	Luo et al. (2020)
RPL18a/eL20	4	89–98	*rpl18ab/+*: male reproductive (stamen) *rpl18ab*: embryo lethal	Xie et al. (2018), Yan et al. (2016)
RPL23A/uL23	2	95	*rpl23aa* knockdown: pointed first leaf, reduced cell division, vascular patterning defects, reduced root growth *rpl23ab* knockdown: no obvious phenotype	Degenhardt and Bonham‐Smith (2008a, 2008b)
RPL23A/uL23	2	95	*rpl23aa*: pointed leaves, retarded root growth, and reduced plant size *rpl23ab*: no obvious phenotype	Xiong et al. (2020)
RPL24/uL24	3	32–93	*rpl24b*: female reproductive (gynoecium) and leaf polarity defects	Nishimura et al. (2005), Yao et al. (2008)
RPL27a/uL15	3	79–97	*rpl27ab*: minor fertility defects *rpl27ac/+*: female reproductive (gynoecium), meristem function, aberrant auxin responses *rpl27ab*, *rpl27ac*: embryo lethal	Szakonyi and Byrne (2011), Zsögön et al. (2014)
RPL28/eL28	2	90	*rpl28a*: leaf polarity defects, needle‐like leaves, meristem defects	Horiguchi et al. (2011), Yao et al. (2008)
RPL36a/eL42	2	100	*rpl36aa* or *rpl36a/+*, *rpl36ab/+ or rpl36ab*: leaf abaxialization defects in *as1* background *rpl36aa*, *rpl36ab*: lethal (female gametophyte); two functional copies required	Casanova‐Sáez et al. (2014)

*Note*: Many RP families appear to act redundantly during development (white) whereas two gene families lack complete redundancy (green). For a number of RP families, only one paralog has been explored, despite differences in the amino acid sequence (blue). RP family size as reported in Hummel et al. (2015).

Unlike in animals, these gametogenesis‐related RP phenotypes are not the result of sex chromosome silencing so are more likely to represent examples of ribosome specialization. However, in many instances, exploring the potential for RP paralogs to contribute to ribosome specialization is complicated by their large gene families (Barakat et al., 2001; Hummel et al., 2015) and not all gene family members have been investigated (Table 2). It is necessary to analyze the expression and mutant phenotypes of other gene family members in order to better understand the contributions of RP paralogs to fertility and gametogenesis.

While most novel phenotypes have been observed in the gametes, it is possible that these phenotypes are actually derived from impaired organ patterning through auxin misregulation. Auxin is a plant hormone that plays a crucial role in defining cellular identity during development. The formation of auxin gradients establishes the apical‐basal axis of the whole plant and individual organs such as the embryo and gynoecium (Berleth et al., 2004). Mutants of auxin‐related genes have been found to exhibit similar phenotypes to those of RP mutants (e.g., vascular defects and aberrant leaf identity) and RP mutants with aberrant auxin responses have been identified (Table 2). Several studies have sought to unravel the connection between RPs and auxin, including application of the GDBH to explain auxin‐related phenotypes (Rosado & Raikhel, 2010). *RPL4D/uL4D* expression has been found to colocalize with auxin maxima and the exogenous application of auxin causes a downregulation of *RPL4/uL4* (Rosado et al., 2010). Early in development *rpl18ab* embryos display diffuse auxin signals, together with mislocalization to the suspensor (Figure 5). These mutants neither maintain suspensor identity (Xie et al., 2018), nor progress past the globular stage of embryogenesis, suggesting that RPL18aB/eL20B helps to maintain the apical‐basal axis during embryo development (Yan et al., 2016) (Figure 5). Whether heterogeneous ribosomal populations have discrete functions in organ pattering is yet to be established.

There is possible reciprocity between mechanisms of translational control by specialized ribosomes and auxin regulation. RPL24B/eL24B has been implicated in ribosome reinitiation to downstream ORFs (Park et al., 2001) (Figure 3(C)) including the auxin‐related genes *ETTIN* (*ETT*) and *MONOPTEROS* (*MP*) (Nishimura et al., 2005). Expression of the main‐ORF (mORF) of *ETT* can partially rescue the *rpl24b* mutant phenotype. This suggests that RPL24B is required for reinitiation to the mORF and that perturbation of this translational mechanism has phenotypic consequences for development of the gynoecium (Nishimura et al., 2005) (Table 1). Similarly, rendering the uORFs of *ETT* nonfunctional results in partial rescue of the *rpl4d/uL4d* and *rpl5a/uL18a* phenotype (Rosado et al., 2012). Consistently, RPL10A/uL1 has also been implicated in uORF‐mediated regulation of translation (Imai et al., 2008). Together, these results indicate that specific RPs may be required for the translational control of specific mRNAs through uORFs (Figure 3(C)).

### 8.3 Multiple paralogs may contribute to ribosome sufficiency rather than specialization

Numerous examples of tissue‐ or cell type‐specific expression of ribosomal paralogs in plants led to the concept that specialized ribosomes could exist and contribute to development (Moin et al., 2016; Weijers et al., 2001; Whittle & Krochko, 2009; Williams & Sussex, 1995). For example, the preferential localization of RPS5B/uS7B to specific regions of the developing embryo (inner cell layers and provascular tissues) (Weijers et al., 2001) raises questions regarding the role of RPS5B/uS7B during embryonic development. In addition, the three *AtRPL10/uL16* paralogs are differentially expressed in response to ultraviolet radiation and in the two gametes (Falcone Ferreyra et al., 2013; Ferreyra et al., 2010).

Although differential paralog expression is indicative of unique functionality, this line of evidence remains controversial. For some RP families, the differential expression of paralogs appears to correlate with the rate of cell division, such that one paralog is highly expressed in cells with high translational requirements and an alternative paralog is more moderately expressed in cells with modest translational requirements. For example, the *RPS5A/uS7A* promoter region is predicted to contain stronger transcriptional elements than that of *RPS5B/uS7B. RPS5A/uS7A* and *RPS16B/uS9B* are strongly expressed in the shoot and root meristems (stem cells). *RPS5B* and *RPS16A/uS9A* are modestly expressed in a subset of nonproliferating but developing tissues, such as the anthers, distal region of the shoot primordium, epidermal cells of the root tip, root hairs and trichomes (leaf hairs) (Weijers et al., 2001; Williams & Sussex, 1995). The correlation with cell division could suggest that paralogs within a gene family are transcribed at different levels so that the rate of paralog transcription changes with the translational requirements of the cell.

Likewise, variable mutant phenotypes do not necessarily equate to unique functionality. Consider the RPL9B, C and D (uL6B‐D) RPs of *A. thaliana* (Table 2). RPL9B and RPL9C proteins are 100% identical, whereas RPL9D shares 89% amino acid identity with B and C. *rpl9d* null mutants are aphenotypic, suggesting the divergent sequence of *RPL9D* does not have functional consequences for development. In contrast, reduced levels of RPL9C (the most highly expressed paralog) yields delayed growth and the classic leaf morphology of *A. thaliana* RP mutants. Reduced levels of *RPL9C* in a *rpl9d* null background results in embryo lethality. Despite different phenotypes resulting from the absence of different paralogs, these data suggest a dosage effect leading to ribosome insufficiency (Devis et al., 2015) rather than functional specialization during development (Table 2). In addition, some mutant phenotypes can be rescued by complementation with another paralog of the same gene family [e.g., *RPL27AA/B* (*uL15A/B*); *RPL9C/D* (*uL6C/D*); *RPL23aA/B* (*uL23A/B*)] suggesting functional redundancy (Devis et al., 2015; Xiong et al., 2020; Zsögön et al., 2014). In the case of the RPL23A/uL23 family, *rpl23aa* displays pointed leaves, retarded root growth and reduced plant size whereas *rpl23ab* appears wild type (Table 2). Rescue of the *rpl23aa* phenotype was achieved by expressing *RPL23AB* under the *RPL23AA* promoter, demonstrating that phenotypic differences are driven by inadequate dosage compensation (Xiong et al., 2020).

### 8.4 Current understanding of RP heterogeneity in plants

Although there is clear evidence that a major driving force behind the retention of RP‐paralogs is to maintain high translational output, there are also hints of unique roles for RPs during development. Understanding why some paralogs are only expressed in a subset of nonproliferating tissues will be key to understanding the extent to which the ribosome sufficiency theory and GDBH explain ribosome heterogeneity. In addition, phenotypic analysis of all members within the gene family is necessary to assess the differential contributions of protein paralogs. Furthermore, many RPs have only been characterized under control conditions but evidence is accumulating for ribosomal heterogeneity during stress response (Martinez‐Seidel et al., 2020). Indeed, differential RP phosphorylation has been observed in response to hypoxia (Bailey‐Serres & Freeling, 1990), flooding (Bailey‐Serres et al., 1997; Bailey‐Serres & Freeling, 1990), and between day and night (Turkina et al., 2011). While the regulatory capacity of RP paralogs is certainly controversial, the remarkable heterogeneity within the plant kingdom is beyond question and yields many exciting possibilities for functional ribosome specialization.

## 9 CONCLUSION

Since 2011 and the reemergence of the theory of specialized ribosomes there has been substantial evidence provided for ribosome heterogeneity. RNA‐Seq analysis indicated that different RP mRNAs were expressed at different time point during development and in different tissues. Cellular proteomics revealed that these transcriptional differences result in changes to protein abundance at the cellular level. In the last few years, more advanced proteomics studies have focused on purified translating ribosomes. Importantly, this revealed that the composition of ribosome itself varies, not just the cellular pool of RPs (Shi et al., 2017). However, there are many outstanding issues that need addressing to gain a more precise understanding of ribosome specialization and how widespread it is:

1. The most significant outstanding question is: Do different ribosome populations actually possess specialized functions? At the center of this is whether these ribosomes translate different mRNA pools. This has been one of the most technically challenging aspects of the field, especially in the context of developmental biology. The Barna group has employed an impressive combination of TRAP‐Seq and Ribo‐Seq to identify which mRNAs different RP‐containing ribosomes are bound to and translating. This level of detail has only been achieved in a very limited setting because the amount of material required for these approaches makes it technically challenging in a developmental setting.
2. If specialized ribosomes exist, then how do they mechanistically affect translation? So far only one mechanism has been characterized and this is the recruitment of specific mRNAs through elements in their 5′‐UTRs via specific RPs to groups of specialized ribosomes. The underlying mechanisms by which specialized ribosomes can regulate specific pools of mRNAs have yet to be discovered. We, and others, have tried to use cryo‐EM to shed light on how ribosome compositional changes might regulate translation but so far this has proved impossible given the technical limitations both dissecting subtle differences in 3D classifications and/or performing EM in purified individual ribosome populations.
3. Are paralogous RPs functionally equivalent? We know that mutations in different members of an RP gene family can generate specific phenotypes, but this does not necessarily mean that the paralogs have disparate functions. Phenotypic differences could arise because of differences in transcript expression between tissues and not because they have different functions. Further work is required to dissect whether paralog‐containing ribosomes translate different mRNAs.
4. Heterogeneous ribosome populations exist, but how are they generated? The most parsimonious theory is that different rRNAs and RPs combinations are generated during ribosome biogenesis. However, as described above, it has been shown that translation of RP mRNAs occurs close to synapses of neurons, a significant distance from the nucleus of the cell (Shigeoka et al., 2019). This suggests that some components could be switched in and out of the ribosome in the cytoplasm, perhaps to allow a rapid response to the environmental stimuli. Work on the dynamics of ribosome composition will be required to understand how widespread these two potential mechanisms are in generating ribosome heterogeneity.
5. Can single ribosomes contain multiple combinations of alternative ribosomal components? Proteomics has been hugely informative in determining that ribosome heterogeneity is widespread but it is not yet understood if there is preference to have multiple alternative components in the same ribosome, that is, does paralog x in one protein preferentially occur with paralog y in another nearby protein. Native mass spectrometry of ribosomes will hopefully provide this important insight (van de Waterbeemd et al., 2018).
6. Does ribosome heterogeneity exist within a cell? Another aspect of heterogeneity not yet fully understood is tissue specificity. Tissue‐specific expression of RPs has been well characterized but what is not yet clear is whether ribosome diversity occurs within a cell rather than in different cells within tissue? Profiling different cell populations within organisms will help address this.

In summary, developmental biology has provided substantial evidence for the specialized ribosome theory from phenotypes and tissue‐specific expression. Recent work has revealed diversity in ribosome composition through RPs, rRNA, modifications, and associated proteins. However, only limited evidence and examples have been discovered that indicate that this heterogeneity represents a potentially exciting new mode of post‐transcriptional regulation. It will be exciting to see how widespread translational regulation by specialized ribosomes turns out to be.

## AUTHOR CONTRIBUTIONS

**Karl Norris:** Writing‐original draft; writing‐review and editing. **Tayah Hopes:** Writing‐original draft; writing‐review and editing. **Julie Aspden:** Writing‐original draft; writing‐review and editing.

## RELATED WIREs ARTICLES

Translation initiation by cap‐dependent ribosome recruitment: Recent insights and open questions

Local translation of mRNAs in neural development

Guarding the ‘translation apparatus’: defective ribosome biogenesis and the p53 signaling pathway
